# Raising Children with Poor School Performance: Parenting Styles and Short- and Long-Term Consequences for Adolescent and Adult Development

**DOI:** 10.3390/ijerph16071089

**Published:** 2019-03-27

**Authors:** Oscar F. Garcia, Emilia Serra

**Affiliations:** Department of Developmental and Educational Psychology, Faculty of Psychology, University of Valencia, Av. Blasco Ibáñez, 21., 46010 Valencia, Spain; emilia.serra@uv.es

**Keywords:** parenting styles, school performance, adolescence, adult development, culture

## Abstract

This study examines the correlates of authoritative (warmth and strictness), indulgent (warmth but not strictness), authoritarian (strictness but not warmth), and neglectful (neither warmth nor strictness) parenting with short- and long-term socialization outcomes in adolescents and adults, with and without poor school performance during adolescence. Short- and long-term socialization outcomes were captured by multidimensional self-esteem (academic/professional, emotional, and family), psychological maturity (self-competence, social competence, and empathy), and emotional maladjustment (nervousness, emotional instability, and hostility). Participants (1195 female and 874 male) consisted of a community sample of adolescents (*n* = 602), young adults (*n* = 610), middle-aged adults (*n* = 469) and older adults (*n* = 388). Design was a 4 × 3 × 2 × 4 MANOVA (parenting style × school performance × sex × age). Results indicated that the relationship between parenting styles and children’s socialization outcomes does not vary as a function of school performance. The link between parenting styles and socialization outcomes shares a common short- and long- term pattern in adolescents and adults: Indulgent parenting was related to equal or even better socialization outcomes than authoritative parenting, whereas authoritarian and neglectful styles were associated with the worst socialization outcomes.

## 1. Introduction

Schools help the children of today to become the adults of tomorrow [1]. Nevertheless, year in and year out, a sizeable proportion of adolescents who do not develop a commitment to succeeding in school or feel of a sense of attachment to school quit before earning their high school diploma [2,3]. Unfortunately, despite public authorities’ efforts to reduce the school dropout rate, this problem remains a pressing public health issue [1,4,5,6,7]. Development during adolescence could be critical (for a review, see Eccles, Midgley, Wigfield, Buchanan, Reuman, Banagan, and Iver, 1994) [8]. The magnitude of the drastic decline in some early adolescents’ school grades as they move into junior high school is a significant predictor of school failure and dropout [9]. Other reductions have been described in adolescent attributes such as academic engagement [10], self-concept and self-perceptions [11,12], interest in school [13], and intrinsic motivation [11]. The relationship between poor academic performance and the dropout rate has been well documented empirically (for a review, see Battin-Pearson, et al., 2000) [3]. Poor academic performance is related to poor self-esteem, especially in the academic and professional domains, and it has a negative impact on the development of psychosocial competence and emotional regulation [3,10,14,15,16,17]. Dropping out of high school may lead to diverse short- and long-term consequences, such as a negative impact on individual well-being, reduced earning potential, and even increased contact with the juvenile and criminal justice systems [18].

Parental socialization has been identified as a major source of protection or risk in childhood, adolescence, and beyond. Parents play a key role in the way their children develop, either contributing to the child’s developmental competence or failing in the parenting socialization process when children manifest a lack of instrumental competence [19,20,21,22]. Nevertheless, the family is not an isolated context where socialization occurs [23,24]. The socialization literature has examined linkages between the child’s family context and his/her school context [14,25,26,27,28]. During adolescence, peer approval may be based less on academic achievement and more on conformity with peer standards that deviate from social norms [29]. For instance, academic engagement and success may be devalued by peers and negatively associated with students’ social standing [30]. Adolescents may also be susceptible to peer pressure about unacceptable behaviors, such as antisocial tendencies [22,31], irresponsible sexual activity [32], or drug use and abuse [33,34]. Despite these extrafamilial influences, parents are still the main socializing agents during adolescence [22,35,36].

To capture parental socialization and its impact on child development, scholars have traditionally followed a four-typology model of parental socialization styles with two orthogonal dimensions: warmth and strictness [20,24,37]. Warmth represents the degree to which parents show their children care and acceptance, support them, and communicate by reasoning with them [20,38]. Other labels such as acceptance [39]; assurance [40]; love [41]; or, more recently, acceptance/involvement [42,43], have similar meanings to warmth. Strictness refers to the degree to which parents impose standards on their children’s conduct, use supervision, and maintain an assertive position of authority over their children. Other labels such as domination; hostility; inflexibility; control; firmness; restriction; or, more recently, strictness/imposition, have similar meanings to strictness [39,41,43,44,45]. Based on these two dimensions, a four-typology classification of child-rearing patterns has been identified: authoritative parents are warm and strict, authoritarian parents are strict but not warm, indulgent parents are warm but not strict, and neglectful parents are neither warm nor strict [20,21,24,37,43,46].

Findings from numerous studies have repeatedly shown the benefits of authoritative parenting (warmth and strictness) as the highest quality parent–child relationship to provide optimal developmental outcomes for children and adolescents from middle-class European-American families [34,43,47]. The positive influence of authoritative parenting has been extended even beyond adolescence; authoritative parenting in childhood and adolescence has been associated with positive functioning in adulthood [48,49,50]. Adolescents from authoritative families develop higher self-esteem [51]; have better psychosocial maturity, as revealed by their strong sense of self-reliance, work-orientation, and social competence [43,52]; report fewer emotional maladjustment problems [43]; have lower rates of drug use and abuse [53,54]; and are less involved in a broad spectrum of behavioral problems [34,43]. Furthermore, authoritative parenting provides various benefits in the school context. Adolescents from authoritative families have good academic competence and orientation toward school, apply the most adaptive achievement strategies (self-enhancing attributions but low levels of failure expectations, task irrelevant behavior, and passivity), achieve better school performance (e.g., grade point average), and are less involved in episodes of school misconduct [25,28,43,52,55]. For example, authoritative parenting is related to the highest school performance, as indicated in many studies examining grade point averages of adolescent students [28,34,36,56]. On the other hand, neglectful parenting (neither warmth nor strictness) is consistently associated with the lowest quality parent–child relationships (the worst developmental outcomes). In the middle position between authoritative (the best) and neglectful (the worst) parenting styles are the authoritarian and indulgent styles. Authoritarian parents (strict but not warm) obtain obedience and conformity with regard to social standards from their children; in an academic context, adolescents from authoritarian parents do well and do not tend to be involved in deviant activities (e.g., school misconduct). However, youngsters with authoritarian parents have relatively worse self-reliance and higher psychosocial and somatic distress. Adolescents with indulgent parents (warm but not strict) show a strong sense of self-confidence, although they fail in an academic context, are less engaged in school, and report more school misconduct [34,43]. In summary, this evidence from studies in middle-class European-American families reveals a repeated pattern of competence and adjustment associated with the four parenting styles: authoritative parenting is the optimal style, neglectful parenting is the worst, and indulgent and authoritarian parenting fall in the middle (e.g., as a mixture of positive and negative traits).

As Pinquart and Kausser recently noted (2018, p. 75) [55], most of the research on the relationship between parenting and children’s psychological and behavioral outcomes has been conducted in middle-class white families from the United States and other western countries. However, the available evidence does not support the idea that the optimal parenting style is always authoritative (warmth and strictness). A growing body of literature questions the view that an authoritative parenting style is always associated with positive developmental outcomes in children across all ethnicities, environments, and cultural contexts [21,57,58,59,60,61,62,63,64,65,66,67,68,69]. Evidence from studies in Anglo-Saxon contexts with ethnic minority families and in cross-cultural parenting research conducted in other cultural contexts casts doubt on whether the warmth (i.e., acceptance and involvement) element of authoritative parenting (shared by authoritative and indulgent parents) is always required for an optimal parenting style [70]. Parenting literature also supports authoritarian parenting (strictness but not warmth) as an appropriate parental strategy in needy ethnic minority families and dangerous communities, where authoritarian parenting may not be as harmful and may even have some protective benefits [71]. For example, when analyzing parenting styles and school context, authoritarian parenting (strictness but not warmth) is associated with optimal academic outcomes and the highest academic grades [42,55,58,72]. Overall, earlier studies in the United States with ethnic minority groups, such as African Americans [57,59,73], Chinese Americans [58,67], Hispanic Americans [74,75], or multiethnic Americans [76], as well as some studies with Arab families, did not find authoritarian parenting to be associated with high levels of psychological distress [60,77,78], suggesting that the authoritarian parenting style is an appropriate parental strategy.

On the other hand, the indulgent parenting style (warmth but not strictness) also provides ample benefits for children’s development in European and Latin American countries, such as Spain [79], Portugal [80], Italy [81], the UK, Sweden, Slovenia, Czech Republic [33], Germany [69], Norway [63], Turkey [82], Brazil [66], or Mexico [83]. Indulgent parenting is related to similar or, in some cases, higher developmental outcomes than authoritative parenting in adolescence. By contrast, both authoritarian parenting (strictness but not warmth) and neglectful parenting (neither warmth nor strictness) are consistently associated with the lowest quality parent–child relationships (the worst developmental outcomes). Some new findings extend the benefits of indulgent parenting beyond adolescence [22,84]. Adolescents from indulgent homes (warmth but not strictness) obtained equal or even higher adjustment than those from authoritative households (warmth and strictness) for different developmental outcomes such as self-esteem [85], psychosocial competence [86], emotional maladjustment [21], substance use and abuse [87], aggression and cyberaggression [88,89], traditional bullying and cyberbullying victimization [31], internalization of values [64,90,91], child-to-parent violence [92], or a broad spectrum of behavioral problems [14,93]. Furthermore, indulgent parenting provides several benefits in the school context. Adolescents from indulgent families have good academic competence, achieve better school performance (e.g., grade point average), report fewer failing grades, and are less involved in episodes of school misconduct. For example, indulgent parenting (warmth but not strictness) helps adolescents in their academic success and school grades [14,21,55,86]. Overall, adolescents with indulgent parents enjoy benefits in the self-reliance domain, as indicated by the positive perceptions of their own personal academic abilities [14,21,86].

### The Present Study

The present study examines the relationship between parenting styles and school performance during adolescence and the pattern of short- and long-term socialization outcomes in adolescents and adults (young, middle-aged, and older adults). Three sets of socialization outcomes will be analyzed: self-esteem, psychosocial maturity, and emotional maladjustment. Self-esteem, psychosocial maturity, and emotional regulation are key goals of socialization [94,95,96]. (i) Self-esteem is a traditional socialization outcome [96] and plays a central role in understanding behavioral, cognitive, emotional, and social functioning in adolescence and adulthood [97,98]. (ii) Psychosocial maturity is another key socialization outcome that represents the response to cultural demands to make an optimal society function [95]. Psychosocial maturity is defined as the capacity “to function effectively on one’s own, or individual adequacy; to interact adequately with others, or interpersonal adequacy; and to contribute to social cohesion, or social adequacy” (Greenberger et al., 1974, p. 128) [95], and it is a key attribute for the optimal growth of the individual associated with positive development in adolescence [43,52,99] and adulthood [100,101,102]. (iii) Emotional maladjustment is a frequent socialization outcome in parenting studies [21,22,86,94], and it represents a failure in the socialization of emotion, where children are not able to regulate their mechanisms of understanding, experiencing, and expressing emotions [94]. Although differences in demographic variables are not central to the focus of parenting studies [21,43], previous research has reported sex- and age-related differences in self-esteem, psychosocial maturity, and emotional maladjustment. Regarding sex-differences, females indicate better academic/professional and family self-esteem but less emotional self-esteem than males. In addition, females have greater psychosocial maturity than males. On emotional maladjustment, females report more nervousness and emotional instability, whereas males indicate more hostility [21,84,86]. Regarding age-related differences, most studies focus on age-specific groups (e.g., adolescents or young adults). Nevertheless, a general tendency suggests that there are age-related increases in self-regulation and reductions in social interest. For example, psychosocial maturity or emotional regulation tends to improve with age [101,103].

Parenting socialization (from childhood to adolescence) is an adult-initiated process (parents or primary caretakers) through which the young person acquires his/her culture, as well as the habits and values congruent with adaptation to that culture, so that young children become responsible members of their society. Unfortunately, when parenting socialization is over, not all children reach the parenting socialization goals and become responsible adult members of their society [19,104]. Despite lifespan development theories that stress the key importance of early experiences well beyond adolescence [105,106], little is known about the links between parenting socialization and psychological and behavioral outcomes in adulthood [49]. In particular, few studies provide evidence about long-term socialization outcomes beyond adolescence [48,49,50,84,107], and most of them have been limited to young adulthood [48,84], used different outcomes for adolescents and for older people [50], or studied isolated parenting practices rather than a parenting styles approach [50,107]. It is commonly recognized that children with low school performance are more likely to have poor psychological competence and consistently worse adjustment on several developmental outcomes. Public health authorities have defended the need for public policies to make a critical contribution to children’s academic achievement [1,3,7]. However, studies commonly use school performance as just another outcome of the parenting style [14,86,108] but not as a public health risk for children that can undermine the adolescent’s development on the crucial path to adulthood; focusing on academic performance as a public health risk would involve analyzing whether the efficacy of parenting is similar or different based on the child’s school performance. For example, previous parenting research has analyzed the impact of parenting in several circumstances, such as raising children in poor neighborhoods [14,109], latchkey children [35], children with antisocial tendencies [84], or even children who are juvenile offenders [110]. Based on the literature review described above, we expect that (1) school performance (medium and high) will be associated with better adjustment (high self-esteem and psychosocial maturity and low emotional maladjustment) than poor school performance (low) and (2) high levels of parental warmth (shared by both authoritative and indulgent parents) will be associated with better socialization outcomes (high self-esteem and psychosocial maturity and low emotional maladjustment) in both the short-term (in adolescents) and long-term (in young, middle-aged, and older adults).

## 2. Materials and Methods

### 2.1. Participants and Procedure

The study was composed of 2069 participants (1195 females and 874 males; *M* = 35.85 years, *SD* = 20.51), 602 adolescents from 12 to 17 years old (351 females and 251 males), 610 young adults from 18 to 35 years old (355 females and 255 males), 469 middle-aged adults from 36 to 59 years old (276 females and 193 males), and 388 older adults from 60 to 75 years old (213 females and 175 males). It was carried out in a south-eastern city of Spain with fewer than one million inhabitants. A priori power analysis determined that 356 participants were required to detect an unfavorable medium effect size (*f* = 0.22) with a power of 0.95 (α = 0.05, 1 − β = 0.95) in *F*-tests among the four parenting styles [111,112]. Data from adolescents and adults were collected during the 2016–2017 and 2017–2018 academic years. (i) Adolescents were recruited from the complete list of high schools through random selection. If a high school refused to participate, a replacement school from the complete list was selected until completing the sample size required. This random sampling procedure means that the probability of each unit in the population (i.e., adolescents from high schools) being selected is the same [21,31,84,113]. To achieve the planned sample size, we contacted the heads of the high schools invited to participate (only two refused to participate). Parental consent was required for adolescent participation. (ii) Young adult participants were recruited in undergraduate education courses, and they received course credit for participating [22,114,115]. (iii) Middle-aged participants were recruited from city council neighborhoods. Three middle-class neighborhoods with similar average household wealth were randomly selected [116,117]. (iv) Older adult participants were recruited from the complete list of senior citizen centers and were randomly selected from the complete list of senior citizen centers. When one senior citizen center refused to participate, another one was selected until completing the sample size required. This system means that every unit in the population (i.e., older adults from senior citizen centers) has the same probability of being selected [21,31,84].

The research protocol was approved by the Research Ethics Committee of the Program for the Promotion of Scientific Research, Technological Development, and Innovation of the Spanish Valencian Region, which supported this research. All the participants who participated in this study (a) were Spanish, as were their parents and four grandparents; (b) lived in two-parent nuclear families with a mother or primary female caregiver and a father or primary male caregiver; and (c) participated voluntarily. A total of 2069 respondents completed the instruments (96% response rate). The power *F*-test among the four parenting styles for the age group with the smallest sample size (older adults, *n* = 388) was 0.95 (*f* = 0.21; α = 0.05) [111,112,118]. All of the questionnaires were completed anonymously with Institutional Review Board approval.

### 2.2. Measures

#### 2.2.1. Parenting Styles

Parental warmth was measured with the 13 items from the warmth/affection scale (WAS) [119]. The WAS measures the extent to which adolescents perceive their parents as loving, responsive, and involved (e.g., “Talks to me about our plans and listens to what I have to say” and “Makes me feel proud when I do well”). The WAS adult version measures the degree to which adults had perceived their parents as loving, responsive, and involved during their adolescence (e.g., “Talked to me about our plans and listened to what I had to say” and “Made me feel proud when I was doing well”). Cronbach’s alpha for this scale was 0.935. Parental strictness was measured using six items from the parental control scale (PCS) [21,33,120]. The PCS measures the extent to which adolescents perceive strict parental control over their behavior (e.g., “They make sure I know exactly what I can and cannot do” and “They believe in having a lot of rules and sticking to them”). The PCS adult version measures the degree to which adults had perceived strict parental control during their adolescence (e.g., “They made sure I knew exactly what I could and could not do” and “They believed in having a lot of rules and sticking to them”). Cronbach’s alpha for this scale was 0.859. On both the WAS and the PCS, adolescents and adults rated all the items on the same 4-point scale from 1 (“almost never true”) to 4 (“almost always true”).

Four parenting styles were defined by dichotomizing the sample on parental warmth and parental strictness and examining the two parenting variables simultaneously [21,33,34,121]: authoritative parenting (above the 50th percentile on both warmth and strictness), neglectful parenting (below the 50th percentile on both variables), authoritarian parenting (above the 50th percentile on strictness, but below the 50th percentile on warmth), and indulgent parenting (above the 50th percentile on warmth, but below the 50th percentile on strictness). The use of the split procedure (e.g., median or tertile) to assign families to the parenting groups, rather than assigning them on the basis of predetermined cutoffs, provides a categorization of families that is sample-specific. For example, families in the “authoritarian” category are indeed relatively more authoritarian (i.e., less warm and stricter) than the other families in the sample, although we do not know whether the families we have labeled “authoritarian” would be considered “authoritarian” within a different population. Therefore, it is important to take into account that the designation of families as one type or another, relative to their counterparts, is done for heuristic, not diagnostic, purposes (see Steinberg et al., 1991, p. 1053) [122].

#### 2.2.2. School Performance

School performance was captured by the grade point average (GPA) in school. Scores were transformed from the Spanish numerical standard (0–10) to the standard GPA in the USA, ranging from 0 (all Fs) to 5 (all As) [43,123]. Adolescent and adult students were asked to report their grade point average (GPA) in the last course in school. Because GPA school records are not always available to students, and there are legal limitations to gaining access to school records in many schools, self-reported GPA is widely used in parenting studies [21,34,36,56]. As Steinberg and Dornsbusch note (1995, p. 917), “self-reported GPA is generally considered to be an accurate assessment of school performance” [34]. In this regard, self-reported grades provide a close approximation to the distribution of grades on school records (see Donovan and Jessor, 1985, 892–893, Dornbusch, Ritter, Leiderman, Roberts, and Fraleigh, 1987, p. 1247–1248) [56,124]. The maximum educational level for participants in the adolescent age group (12 to 17 years old) was the compulsory secondary education certificate, whereas for young adults (18 to 35 years old), middle-aged adults (36 to 59 years old), and older adults (60 to 75 years old), it was a doctorate degree. Each participant was categorized into low, medium, and high performance in school based on a tertile split within their sex and age peer group (adolescent, young, middle-aged, or older adults), reflecting their relative standing within their age peer group [125,126].

#### 2.2.3. Self-Esteem

Academic/professional, emotional, and family self-esteem were measured with three 6-item subscales from the multidimensional self-esteem scale (AF5) [97,127,128]. The AF5 is a widely validated questionnaire for adolescents and adults [97,117,128,129,130,131] in several countries such as Spain [129,131], Portugal [130], Brazil [97], Chile [117], and the United States [128]. The academic/professional component denotes the perception that adolescents or adults have of the quality of their role performance as students (or workers). A sample item is “I work very hard in class [at work]”. The emotional component denotes the perception that adolescents or adults have of their emotional state and their responses to specific situations, with some degree of commitment and involvement in their daily lives. A sample item is “I am afraid of some things” (reversed item). The family component refers to the perception that adolescents or adults have of their involvement, participation, and integration in the family. A sample item is “My family is disappointed with me” (reverse item). Participants responded on a 99-point scale, ranging from 1 (strong disagreement) to 99 (strong agreement). Modifications were made to obtain a score index ranging from 0.10 to 9.99. Higher scores represent a greater sense of self-esteem. Cronbach’s alpha for each subscale was academic/professional, 0.880; emotional, 0.757; and family, 0.810.

#### 2.2.4. Psychosocial Maturity

Psychosocial maturity was measured with the self-competence, social competence, and empathy subscales of the psychosocial maturity questionnaire (CRPM3) [22,43,99]. Self-competence was measured with 12 items. Two sample items are “I consider myself effective in my work” and “I have confidence and security in myself”. Social competence was measured with eight items. Two sample items are “I adapt successfully to different people and social situations” and “I am able to maintain very close ties of friendship with others”. Empathy was measured with five items. Two sample items are “I am sensitive to others’ feelings and needs” and “I know how to listen to other people”. On all subscales, adults responded on a 5-point scale ranging from 1 (strongly disagree) to 5 (strongly agree). Higher scores on self-competence, social competence, and empathy represent a greater sense of psychosocial maturity. Cronbach’s alpha value for each subscale was self-competence, 0.860; social competence, 0.831; and empathy, 0.672.

#### 2.2.5. Emotional Maladjustment

Emotional maladjustment was measured with the nervousness, emotional instability, and hostility subscales. Nervousness was assessed with eight items from the CRPM3 [22,43,99]. Two sample items are: “I am usually tense, nervous and anxious” and “I get irritated easily”. Participants responded on a 5-point scale ranging from 1 (strongly disagree) to 5 (strongly agree). Higher scores on nervousness represent greater emotional maladjustment. Cronbach’s alpha value was 0.775. Emotional instability and hostility were assessed with the two subscales of the Personality Assessment Questionnaire (PAQ) [21,86,132]. Emotional instability was assessed with six items. Two sample items are “I am in a bad mood and grouchy without any good reason” and “I am cheerful and happy one minute and gloomy or unhappy the next”. Hostility was assessed with six items. Two sample items are “I think about fighting or being mean” and “I get so mad I throw or break things”. Participants responded on a 4-point scale ranging from 1 (almost never true) to 4 (almost always true). Higher scores on instability and hostility represent greater emotional maladjustment. Cronbach’s alpha for each subscale was emotional instability, 0.711; and hostility, 0.659.

### 2.3. Data Analyses

A factorial (4 × 3 × 2 × 4) multivariate analysis of variance (MANOVA) was applied for three sets of socialization outcome variables (self-esteem, psychosocial maturity, and emotional maladjustment), with parenting style (authoritative, authoritarian, indulgent, and neglectful), school performance (low, medium, and high), sex (male vs. female), and age group (adolescents, young adults, middle-aged adults, and older adults) as independent variables. Follow-up univariate *F*-tests were performed for all sources of variation when multivariate statistically significant differences were found. Univariate significant results were followed by post-hoc Bonferroni comparisons of all the possible pairs of means [21,34,43,80].

## 3. Results

### 3.1. Parenting Style Groups

Participants were classified into one of four parenting typologies (indulgent, authoritative, authoritarian, or neglectful) (Table 1). The indulgent group contained 577 children (27.9%) with high warmth, *M* = 73.71, *SD* = 4.45, but low strictness, *M* = 28.17, *SD* = 5.54; the authoritative group contained 451 (21.8%) with high warmth, *M* = 72.82, *SD* = 4.18, and high strictness, *M* = 39.87, *SD* = 5.13; the authoritarian group contained 591 (28.6%) with low warmth, *M* = 55.35, *SD* = 10.02, and high strictness, *M* = 41.95, *SD* = 5.76; and the neglectful group contained 450 (21.7%) with low warmth, *M* = 57.35, *SD* = 9.29, and low strictness, *M* = 28.28, *SD* = 5.59. In agreement with the orthogonality assumption, the warmth and strictness parental dimensions were weakly intercorrelated across the four age groups: 12–17 years, *r* = −0.203, *R*^2^ = 0.04, 95% CI (0.08, 0.02), less than 5% of shared variance, *p* < 0.005; 18–35 years, *r* = −0.202, *R*^2^ = 0.04, 95% CI (.08, 0.02), less than 5% of shared variance, *p* < 0.005; 36–59 years, *r* = −0.209, *R*^2^ = 0.04, 95% CI (0.09, 0.01), less than 5% of shared variance, *p* < 0.005; and 60–75 years, *r* = −0.216, *R*^2^ = 0.05, 95% CI (0.10, 0.01), 5% of shared variance, *p* < 0.005. The distribution of the parenting styles by sex was homogeneous, χ^2^(3) = 0.48, *p* = 0.923, as was their distribution by age, χ^2^(3) = 1.96, *p* = 0.992. In the group of authoritative families, there were 451 participants (31.04% adolescents, 29.27% young adults, 22.39% middle-aged adults, and 17.29% older adults). In the group of indulgent families, there were 577 participants (28.77% adolescents, 29.12% young adults, 23.33% middle-aged adults, and 18.22% older adults). In the group of authoritarian families, there were 591 participants (28.09% adolescents, 29.95% young adults, 22.50% middle-aged adults, and 19.46% older adults). In the group of neglectful families, there were 450 participants (28.89% adolescents, 29.56% young adults, 23.33% middle-aged adults, and 18.22% older adults).

### 3.2. Multivariate Analyses

The four MANOVA main effects were statistically significant for parenting style, Λ = 0.759, *F*(27, 5751.1) = 21.09, *p* < 0.001, school performance, Λ = 0.980, *F*(18, 3938.0) = 10.83, *p* < 0.001, sex, Λ = 0.888, *F*(9, 1969.0) = 27.57, *p* < 0.001, and age Λ = 0.830, *F*(27, 5751.1) = 14.00, *p* < 0.001 (Table 2). In addition; the MANOVA analysis yielded statistically significant interaction effects between parenting style and age, Λ = 0.933, *F*(81, 12,733.7) = 1.69, *p* <0.001, school performance and sex, Λ = 0.985, *F*(18, 3938.0) = 1.66, *p* = 0.039, school performance and age, Λ = 0.938, *F*(54, 10,044.6) = 2.35, *p* < 0.001, and sex and age, Λ = 0.979, *F*(27, 5751.1) = 1.52, *p* = 0.042.

### 3.3. Parenting Styles and Self-Esteem Outcomes

**Figure 1:**
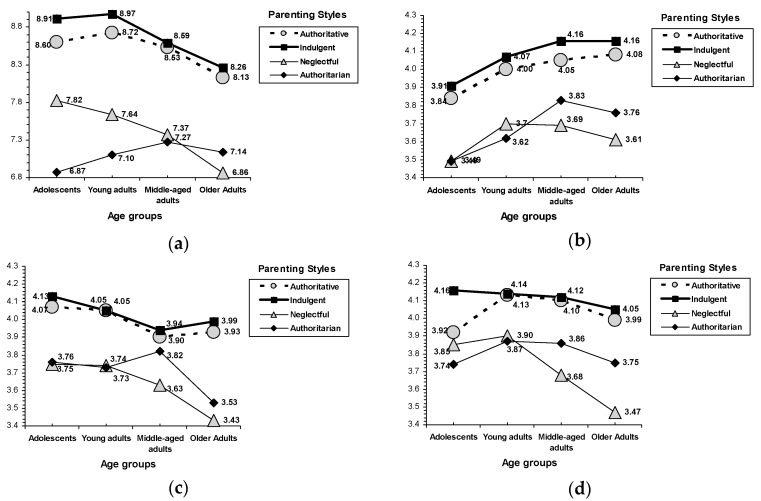
Interactions for parenting style by age. (**a**) Family self-esteem, (**b**) self-competence, (**c**) social competence, and (**d**) empathy.

Indulgent parenting was associated with equal or even higher self-esteem than the authoritative style; by contrast, authoritarian and neglectful parenting were always related to the lowest level of self-esteem (Table 3). On academic/professional self-esteem, children with indulgent and authoritative parents obtained higher scores than those from authoritarian and neglectful families. On emotional self-esteem, indulgent parenting was related to higher scores than the authoritative, authoritarian, and neglectful styles. Similarly, an interaction effect between parenting styles and age was found on family self-esteem, *F*(9, 1977) = 3.69, *p* < 0.001 (see Figure 1). Again, indulgent and authoritative parenting styles were more related to higher family self-esteem than neglectful and authoritarian parenting in adolescents and adults. Age profiles showed a drastic decrease in family self-esteem within neglectful parenting (older adults raised in neglectful families reported lower scores than adolescents and young adults who characterized their parents as neglectful). Of the parenting styles related to low family self-esteem (i.e., neglectful and authoritarian), neglectful parenting was associated with higher scores than the authoritarian style but only in the adolescent and young adult age groups; in middle-aged and older adults, scores were not statistically different.

### 3.4. Parenting Styles and Psychosocial Maturity Outcomes

Again, indulgent parenting was associated with equal or even better psychosocial maturity than authoritative parenting, whereas the lowest psychosocial maturity scores corresponded to authoritarian and neglectful parenting. An interaction effect between parenting styles and age was found on self-competence, *F*(9, 1977) = 2.48, *p* = 0.008; social competence, *F*(9, 1977) = 1.95, *p* = 0.042; and empathy, *F*(9, 1977) = 2.85, *p* = 0.002 (see Figure 1). On self-competence, age profiles indicated that the indulgent and authoritative styles were related to higher scores than the neglectful and authoritarian styles in adolescents and adults (young, middle-aged, and older adults). For the parenting styles related to poor self-competence (i.e., neglectful and authoritarian), differences between the two parenting styles did not reach statistical significance in any age group. On social competence, adolescents and adults from indulgent and authoritative families reported higher scores than those from authoritarian and neglectful households (although in the middle-aged adult group, parenting differences only reached statistical levels between the indulgent and neglectful styles). A general lower tendency related to age was found (e.g., older adults had lower social competence than adolescents and young adults). However, this decreasing tendency was especially salient in parenting styles characterized by lack of warmth (i.e., authoritarian and neglectful). As family age profiles revealed, in participants from neglectful families, older adults reported lower scores than adolescents and young adults; and in those from authoritarian households, older adults reported lower scores than middle-aged adults. On empathy, indulgent parenting was related to better scores than authoritative parenting in the adolescent age group. The poorest empathy scores corresponded to the authoritarian and neglectful styles. For empathy, similar to social competence, the age profile showed a drastic decreasing tendency with age in children from neglectful families (older adults reported lower scores than adolescents and young adults).

### 3.5. Parenting Styles and Emotional Maladjustment Outcomes

Overall, indulgent parenting was consistently associated with less emotional maladjustment than the authoritative, authoritarian, and neglectful parenting styles (see Table 3). On nervousness, children from indulgent families obtained the lowest scores, whereas the highest scores corresponded to authoritarian and neglectful parenting, and authoritative parenting was in the middle position. For emotional instability, the indulgent parenting style was associated with lower scores than authoritative, authoritarian, and neglectful parenting (authoritarian parenting was related to higher scores than authoritative parenting). In the case of hostility, children from indulgent families obtained lower scores than those from authoritative families, whereas children from authoritarian and neglectful households indicated the highest hostility scores.

### 3.6. School Performance

**Figure 2:**
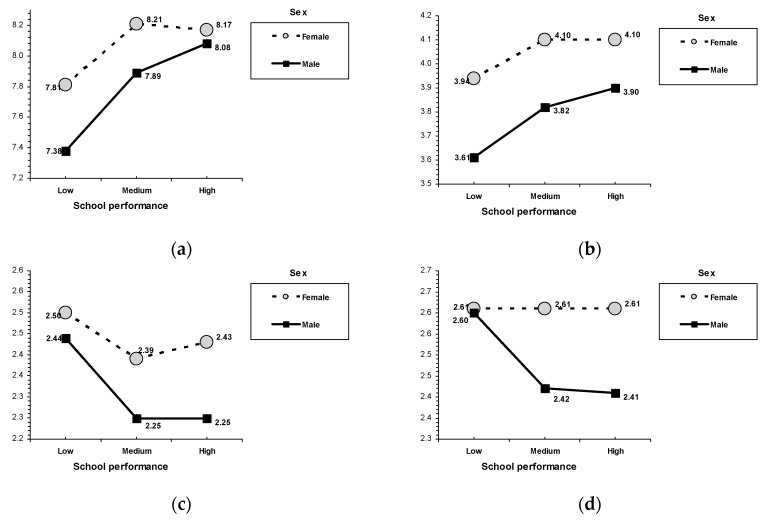

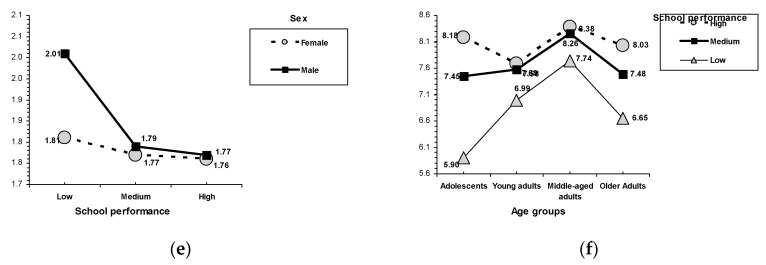
Interactions for school performance and sex. (**a**) Family self-esteem, (**b**) empathy, (**c**) nervousness, (**d**) emotional instability, and (**e**) hostility. Interactions for school performance and age. (**f**) Academic/professional self-esteem.

Results indicated that poor school performance was associated with the lowest self-esteem and psychosocial maturity and the highest emotional maladjustment (see Table 3). For self-esteem, poor school performance was related to the lowest levels of academic/professional and family self-esteem. An interaction effect between school performance and age was found on academic/professional self-esteem, *F*(6, 1977) = 8.32, *p* < 0.001 (see Figure 2). In the adolescent age group, low school performance was related to the lowest academic/professional self-esteem, whereas high performance in school was associated with the highest scores (medium school performance was in the middle position). In the adult age groups, results indicated that young, middle-aged, and older adults with poor school performance during their adolescence reported lower academic/professional self-esteem in adulthood than those with medium and high performance in school. In the case of family self-esteem, low school performance was associated with lower scores than medium and high performance in school. In a similar way, for psychosocial maturity, low school performance was related to lower self-competence and empathy than medium and high performance in school. On emotional maladjustment, poor school performance was associated with the highest levels of nervousness, emotional instability, and nervousness.

### 3.7. Sex and Age

**Figure 3:**
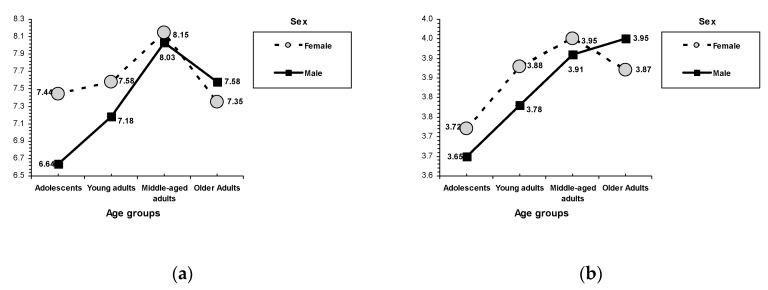
Interactions between sex and age. (**a**) Academic self-esteem and (**b**) self-competence.

Although not the focus of this study, several univariate main effects for sex and age attained significance (see Table 4). Sex-related differences indicated that females had more academic/professional and family self-esteem but less emotional self-esteem than males. An interaction effect between school performance and sex was found on family self-esteem, *F*(2, 1977) = 3.38, *p* = 0.034 (see Figure 2), such that females with poor school performance reported higher scores than males with poor school performance. On psychosocial maturity outcomes, females showed more empathy and social competence than males. An interaction effect between school performance and sex was found on empathy, *F*(2, 1977) = 3.71, *p* = 0.025, with females reporting higher empathy than males (regardless of school performance). In the case of emotional maladjustment outcomes, an interaction effect between sex and school performance was found on nervousness, *F*(2, 1977) = 3.09, *p* = 0.046; emotional instability, *F*(2, 1977) = 5.65, *p* = 0.004; and hostility, *F*(2, 1977) = 6.77, *p* = 0.001 (see Figure 3). Males with medium and high performance in school reported lower nervousness and emotional instability than females with the same school performance (no sex differences were found within the poor school performance condition). On hostility, only in the low school performance condition, males reported higher scores than females. 

Age-related differences were found in all the socialization outcomes. On academic/professional self-esteem, adolescents had lower scores than the adult age groups (the peak corresponded to middle-aged adults); on emotional self-esteem, older and middle-aged adults showed higher scores than adolescents and young adults; and on family self-esteem, the lowest scores corresponded to older adults. An interaction effect between age and sex was found on academic/professional self-esteem, *F*(6, 1977) = 6.49, *p* < 0.001 (see Figure 2). In the adolescent and young adult age groups, females obtained higher scores than males. On psychosocial maturity, adolescents showed lower self-competence than adults; older adults showed lower social competence than adolescents and young adults; and young adults obtained the highest empathy. An interaction effect between age and sex was found on self-competence, *F*(3, 1977) = 2.35, *p* = 0.070 (see Figure 2). Both males and females showed increased self-competence related to age (middle-aged adults scored higher than adolescents). Older male adults scored higher than middle-aged male adults, whereas older female adults scored lower than middle-aged female adults (although these differences did not reach statistical significance).

## 4. Discussion

This study examines the links between parenting styles and school performance during adolescence and short- and long-term socialization outcomes in a community sample of Spanish adolescents and adults (young, middle-aged, and older adults). The short- and long-term socialization outcomes analyzed were self-esteem (academic/professional, emotional, and family), psychosocial maturity (self-competence, social competence, and empathy), and emotional maladjustment (nervousness, emotional instability, and hostility). We examine whether consequences of parenting styles for children’s socialization outcomes could be different depending on school performance. Overall, an important contribution of this study is that our results did not reveal an interaction between parenting style and school performance; therefore, the relationship between parenting styles and children’s socialization outcomes does not vary based on school performance. In general, results indicated that the indulgent style (warmth but not strictness) is an effective parenting strategy, regardless of the child’s school performance. Children raised in indulgent families obtained equal or even higher competence and adjustment than those who were raised in authoritative households. Both authoritarian and neglectful parenting (lack of warmth) were related to the worst outcomes. Moreover, it is important to note that poor school performance is consistently associated with the worst short- and long-term socialization outcomes, not only during adolescence but also in adulthood.

On the self-esteem outcomes, our results indicated that indulgent parenting is associated with equal (academic/professional and family) or even higher (emotional) levels of self-esteem. By contrast, authoritarian and neglectful parenting styles were consistently related to the lowest levels of self-esteem (academic/professional, emotional, and family). Additionally, the parenting age profile for family self-esteem indicated that, despite a decreasing tendency related to age (e.g., older adults reported the lowest family self-esteem), both adolescents and adults (young, middle-aged, and older adults) from indulgent and authoritative families reported more family self-esteem than those from neglectful and authoritarian households. This decreasing tendency was especially salient within the neglectful style; older adults who were raised by neglectful parents reported lower family self-esteem than adolescents and young adults who characterized their parents as neglectful. Again, on psychosocial maturity outcomes, a similar parenting age profile was found; indulgent and authoritative parenting styles were related to greater self-competence, social competence, and empathy than authoritarian and neglectful parenting. Interestingly, the parenting age profile revealed a different pattern between families characterized by high warmth (indulgent and authoritative) and families characterized by low warmth (authoritarian and neglectful). A decreasing tendency related to age was found, but only in children from neglectful families (older adults reported lower social competence and empathy than adolescents and young adults) and children from authoritarian households (older adults reported lower social competence than middle-aged adults). Furthermore, indulgent parenting was related to more empathy than authoritative parenting in the adolescent age group. Finally, the indulgent parenting style was consistently associated with the lowest levels of emotional maladjustment. Children from indulgent families reported lower nervousness, emotional instability, and hostility than their counterparts from authoritative households. Authoritative parenting was related to less emotional nervousness than authoritarian parenting, and less emotional instability than neglectful parenting.

Another main contribution of our study is that the present results show the linkage between parenting styles and socialization outcomes in the short and long term for three socialization outcomes: self-esteem, psychosocial maturity, and emotional maladjustment. Our results support the idea suggested by earlier socialization researchers [34,57]; that is, the benefits of optimal parenting tend to maintain high adjustment, whereas the deleterious consequences of the worst parenting tend to accumulate over time [49,50,107]. The present findings show that for both adolescents and adults (young, middle-aged, and older adults), the indulgent parenting style is related to optimal short- and long-term socialization outcomes (the highest self-esteem and psychosocial maturity and the lowest emotional maladjustment). Therefore, our findings show that high levels of parental acceptance and involvement combined with low levels of strictness and imposition (i.e., indulgent parenting) seem to make up an optimal parenting strategy in the European cultural context, thus confirming and extending results from previous studies conducted in European and South American countries [21,31,33,62,86]. Self-esteem, psychosocial maturity, and emotional regulation are key goals of socialization [94,96,99]. Results of this study contrast with findings from other cultural contexts where a high level of parental strictness is the key component in fostering the development of children’s self-esteem, psychosocial maturity, and emotional regulation [43,52]. Compared to research conducted mainly in Anglo-Saxon countries, in this study with a European community sample of adolescents and adults, we found that parental warmth and involvement (common in authoritative and indulgent families), rather than parental strictness and imposition (common in authoritative and authoritarian styles), are key strategic factors in promoting the offspring’s developmental competence and adjustment. Moreover, the strictness component not only seems to be superfluous but it could also be negative in the short- and long- term developmental competence of adolescents and adults (authoritative parenting was related to less emotional self-esteem and more emotional maladjustment than indulgent parenting).

The present work also addressed main gaps in previous findings examining the linkage between parenting styles and short- and long-term socialization outcomes. Most of the previous studies examining long-term socialization outcomes have only focused on young adults [22,48]. Four other limitations of previous parenting studies should be noted. First, they used different short- and long-term socialization outcomes for adolescents and for older people [50]. Second, even when the socialization outcomes were the same, the study was limited to adolescents and older adults [84]. Third, they used specific age groups of adult children (e.g., 36, 46, and 60–64 years old) rather than global adult age groups [50]. Four, they examined isolated parenting practices rather than using a parenting style approach [50,107]. By contrast, our study provides evidence through a parenting styles framework that captures overall long-term parenting characteristics that integrate and organize particular or specific parenting practices. Furthermore, the impact of parenting styles was analyzed by examining the relationships between parenting styles and children’s short- and long-term adjustment or maladjustment, using the same set of socialization outcomes (self-esteem, psychosocial maturity, and emotional maladjustment) and nine indicators for adolescents and adults. The results confirm previous results about children’s short-term adjustment in the Spanish context [21,86], but they also extend evidence to the classical adult age groups (young, middle-aged, and older adults) widely used in adulthood studies [133].

Although a main contribution of this study is that the relationship between the parenting style and the outcomes does not vary depending on school performance, it is crucial to note that the present findings corroborate those of other scholars and expand previous work by showing the key role of experiences in the school context in competence and personal adjustment in adolescence and beyond. Analyzing the main effects, the results showed that, in adolescents and adults (young, middle-aged, and older adults), poor school performance (low) during adolescence was consistently associated with the worst outcomes: less self-esteem (academic/professional and family), less psychosocial maturity (self-competence and empathy), and greater emotional maladjustment (nervousness, emotional-instability, and hostility). Although adolescence ends for all adolescents, developmental progress into healthy adulthood is not guaranteed for all. As our results show, adolescents but also adults’ with poor school performance during adolescence had lower competence and adjustment levels. We found differences in all three socialization outcomes and in seven of the nine criteria. Importantly, the negative impact of poor school performance is not limited to the academic or professional domain (e.g., self-perceptions or lack of individual adequacy); instead, the harm extends to other relevant competences, such as self-esteem, psychosocial maturity, and emotional regulation. For example, adolescents and adults with poor school performance during adolescence have lower family self-esteem, less empathy, and greater emotional instability. Our findings contradict some previous studies supporting the idea that a certain degree of discomfort, disruptiveness, and defiance may be normative in adolescence because adolescents have to free themselves from dependence on their parents to form an identity of their own on the path to healthy adulthood [100,134]. Therefore, these results do not confirm the so-called classic “storm and stress” hypothesis (for a review, see Arnett, 1999) [135]. On the one hand, our results agree with previous studies supporting the idea that adolescents who do not fit social standards (e.g., those with antisocial behavior) fail in their developmental progress into healthy adulthood [22,136], extending the evidence to academic standards. In this regard, the present findings revealed that adolescents who do not meet academic standards (e.g., those with poor school performance) suffer incompetence and maladjustment in adulthood. As expected, although the present results indicate a general negative impact of poor school performance on competence and adjustment; the greatest variations in competence and adjustment that differentiate successful (i.e., medium and high performance in school) from unsuccessful students (i.e., poor school performance) lie in the realm of self-perceptions and psychosocial maturity, particularly academic/professional self-esteem and self-competence [52,99,137].

Furthermore, results of this study agree with previous findings on the relations between the demographic variables of sex and age and competence and adjustment. Overall, females showed the highest family self-esteem and academic/professional self-esteem, whereas males reported more emotional self-esteem than females. Females reported more empathy and social competence than males. Males reported more hostility, and females reported more nervousness and emotional instability [21,84,86]. These results also offer age differences that agree with some scholars who suggest age-related increases in self-regulation and reductions in social interest, as well as a peak in the professional career in middle adulthood [101,103,138]. Overall, academic/professional self-esteem was higher in adults than in adolescents (the peak corresponded to middle-aged adults); older and middle-aged adults reported higher emotional self-esteem than young adults and adolescents; and older adults reported the lowest levels of family self-esteem. Adolescents reported lower self-competence than adults, older adults indicated the lowest levels of social competence, and young adults indicated the highest empathy. In terms of emotional maladjustment, adolescents indicated the highest levels of emotional instability and hostility.

This study has strengths and limitations. The two-dimensional four-style theoretical framework to assess parenting offers the opportunity to examine parenting across the globe by examining parenting styles in the broad context of different outcomes through different demographic variables, settings, and countries, contributing to the replication and consistency of the empirical evidence. The present study, with a cross-sectional design, does not determine a relationship of causality between variables, and it cannot exclude other third variables (e.g., there is a long time lag between the parenting socialization and the older adults’ current development), although it establishes linkages between parenting styles and adolescents’ school performance and short- and long-term socialization outcomes in Spanish adolescents and adults (young, middle-aged, and older adults). These findings should be interpreted with some caution because we cannot exclude either causal relations between variables or third-variable explanations, but the relative demographic similarity of the sample makes such third-variable accounts less likely. Participants reported their parents’ behavior [34], although similar results have been obtained in parenting style studies, despite different methods of data collection (e.g., data provided by parents or by external observers) [34,43,139,140]. In the absence of longitudinal or experimental data, the findings must be viewed as preliminary. Finally, this study uses a community sample, rather than an ethnic minority or clinical sample, although the results offer evidence consistent with previous research. More studies are needed with other samples, such as people from poor neighborhoods or other cultural contexts, in order to extend the parenting evidence, particularly about whether the relations between parenting styles and socialization outcomes may vary as a function of school performance.

As socialization theorists explain, modern societies cannot rely on the ubiquitous presence of policemen or monitors (e.g., parents or caretakers) to keep individual members of society in line [104]. There comes a time when parenting socialization is over: the child has become an adult. However, as in childhood and adolescence, our results show that there are theoretically predictable differences in competence and adjustment among adults who were raised in authoritative, authoritarian, indulgent, and neglectful homes (despite the many variables affecting development in adulthood). Adults who were raised by indulgent families have the best competence and adjustment in terms of self-esteem, psychosocial maturity, and emotional regulation. The present results imply that adolescence may represent the last opportunity for parenting socialization; therefore, as other scholars pointed out, it is of interest to test what the optimal style is for parents with adolescent children who not fit social or academic standards. For example, Steinberg and colleagues (2006) [110] test whether there would be theoretically predictable differences among adolescents who do not fit the social standards (serious juvenile offenders) from authoritative, indulgent, authoritarian, and neglectful families, in order to identify the optimal parenting style. Future studies should more thoroughly examine the correlates of parenting styles among adolescents who are at the greatest risk of developmental progress into unhealthy adulthood [141,142,143]. Additionally, our study has other important implications in the family field because it provides insights to orient parental education programs that could improve relationships with children (not only adolescents, even adults) and enhance their psychological and social resources, well-being, and quality of life.

## 5. Conclusions

Finally, the findings of the present study agree with conceptions from recent parenting literature about children’s poor school performance as a pandemic community problem, offering and discussing alternative views of the normative function of children’s poor school performance during adolescence. Currently, the World Health Organization (2014, p. 8) [7] warns that it is crucial to pay more attention to the health-compromising behaviors and conditions that arise during adolescence and can have a long-term impact on health across the lifetime. In this regard, the present study revealed that, although there can be adolescence-limited decreases in academic competence, the majority of Spanish adolescents with poor school performance have several different indicators of maladjustment during adulthood. Before implementing and developing public policies and laws that facilitate and mandate interventions in order to protect adolescents from harm, it is important to identify commonality among risk and protective factors in the family context. Our study, which agrees with a growing set of studies in Europe and South America, indicates that indulgent parenting (warmth but not strictness) is the optimal strategy and is associated with better short-term and long-term outcomes than authoritative parenting (warmth and strictness). Therefore, parental warmth is consistently a protective factor, whereas strictness does not offer protection and could even be associated with harm, highlighting the importance of the cultural context in which parental socialization takes place.

## Figures and Tables

**Table 1:** Numbers of cases in parenting style groups, mean scores, and standard deviations for main measures of parental dimensions.

	Total	Authoritative	Indulgent	Authoritarian	Neglectful
Frequency	2069	451	577	591	450
Percent	100	21.8	27.9	28.6	21.7
Warmth					
*Mean*	67.72	72.82	73.71	55.35	57.35
*SD*	11.42	4.18	4.45	10.02	9.29
Strictness					
*Mean*	34.68	39.87	28.17	41.95	28.28
*SD*	8.50	5.13	5.54	5.76	5.59

**Table 2:** Four-way multivariate analysis of variance (MANOVA) factorial 4 × 3 × 2 × 4 for the three sets of outcomes measures: self-esteem, psychosocial maturity, and emotional maladjustment.

Source of Variation	Λ	*F*	*df*_between_	*df*_error_	*p*
(A) Parenting Styles ^a^	0.759	21.09	27	5751.1	<0.001
(B) School performance ^b^	0.980	10.83	18	3938.0	<0.001
(C) Sex ^c^	0.888	27.57	9	1969.0	<0.001
(D) Age ^d^	0.830	14.00	27	5751.1	<0.001
A × B	0.972	1.05	54	10,044.6	0.373
A × C	0.979	1.38	27	5751.1	0.090
A × D	0.933	1.69	81	12,733.7	<0.001
B × C	0.985	1.66	18	3938.0	0.039
B × D	0.938	2.35	54	10,044.6	<0.001
C × D	0.979	1.52	27	5751.1	0.042
A × B × C	0.974	0.96	54	10,044.6	0.560
A × B × D	0.917	1.05	162	15,964.9	0.305
A × C × D	0.961	0.97	81	12,733.7	0.561
B × C × D	0.980	0.88	45	8810.9	0.696
A × B × C × D	0.930	1.07	135	15,334.8	0.283

^a^*a*_1_, authoritative, *a*_2_, indulgent, *a*_3_, authoritarian, *a*_4_, neglectful; ^b^*b*_1_, low, *b*_2_, high, *b*_3_, high; ^c^*c*_1_, male, *c*_2_, female; ^d^*d*_1_, adolescents (12–17 years), young adults (18–35 years), middle-aged adults (36–59 years), and older adults (60–75 years).

**Table 3:** Means (and standard deviations) for parenting style and school performance, and main univariate *F* values for the set of outcome measures (self-esteem, psychosocial maturity, and emotional maladjustment).

Socialization Outcomes	Parenting Style					School Performance			
	Authoritative	Indulgent	Authoritarian	Neglectful	*F*(3, 1977)	Low	Medium	High	*F*(3, 1977)
Self-esteem									
Academic/professional	7.82 ^1^	7.92 ^1^	7.09 ^2^	7.12 ^2^	33.42 ***	6.82 ^3^	7.67 ^2^	8.01 ^1^	81.65 ***
	(1.35)	(1.20)	(1.68)	(1.51)		(1.78)	(1.22)	(1.17)	
Emotional	5.59 ^2^	5.95 ^1^	5.39 ^2^	5.63 ^2^	8.04 ***	5.60	5.52	5.86	0.81
	(1.78)	(1.82)	(1.71)	(1.67)		(1.68)	(1.77)	(1.81)	
Family	8.54 ^1^	8.73 ^1^	7.08 ^2,b^	7.49 ^2,a^	150.16 ***	7.60 ^2^	8.12 ^1^	8.12 ^1^	9.00 ***
	(1.02)	(0.94)	(1.58)	(1.45)		(1.53)	(1.38)	(1.44)	
Psychosocial maturity									
Self-competence	4.00 ^1^	4.04 ^1^	3.66 ^2^	3.62 ^2^	65.80 ***	3.67 ^2^	3.88 ^1^	3.95 ^1^	21.98 ***
	(0.49)	(0.49)	(0.59)	(0.57)		(0.69)	(0.64)	(0.68)	
Social-competence	4.00 ^1^	4.04 ^1^	3.72 ^2^	3.66 ^2^	36.56 ***	3.78	3.93	3.86	1.14
	(0.58)	(0.62)	(0.70)	(0.68)		(0.69)	(0.64)	(0.68)	
Empathy	4.03 ^1^	4.12 ^1^	3.81 ^2^	3.76 ^2^	45.41 ***	3.78 ^2^	4.02 ^1^	3.99 ^1^	15.58 ***
	(0.70)	(0.65)	(0.71)	(0.66)		(0.63)	(0.54)	(0.54)	
Emotional maladjustment									
Nervousness	2.29 ^2^	2.18 ^3^	2.57 ^1^	2.50 ^1^	26.01 ***	2.47 ^1^	2.35 ^2^	2.34 ^2^	4.62 *
	(0.61)	(0.63)	(0.64)	(0.61)		(0.66)	(0.62)	(0.65)	
Emotional-instability	1.79 ^1,b^	1.67 ^2^	1.92 ^1,a^	1.90 ^1^	9.04 ***	2.61 ^1^	2.55	2.50 ^2^	3.77 *
	(0.47)	(0.42)	(0.53)	(0.49)		(0.55)	(0.56)	(0.57)	
Hostility	2.56 ^2^	2.44 ^3^	2.65 ^1^	2.57 ^1^	21.03 ***	1.91 ^1^	1.78 ^2^	1.76 ^2^	6.76 **
	(0.47)	(0.44)	(0.56)	(0.47)		(0.53)	(0.45)	(0.48)	

* *p* < 0.05; ** *p* < 0.01; *** *p* < 0.001; ^#^ α = 0.05; ^1^ > ^2^ > ^3^ > ^4^; ^a^ > ^b^.

**Table 4:** Means (and standard deviations) for parenting style and school performance, and main univariate *F* values for the set of outcome measures (self-esteem, psychosocial maturity, and emotional maladjustment).

Socialization Outcomes	Sex			Age				
	Female	Male	*F*(1, 1977)	12–17 Years	18–35 Years	36–59 Years	60–75 Years	*F*(1, 1977)
Self-esteem								
Academic/professional	7.63	7.29	8.51 **	7.10 ^3^	7.41 ^2^	8.10 ^1^	7.45 ^2^	38.81 ***
	(1.43)	(1.57)		(1.59)	(1.36)	(1.19)	(1.66)	
Emotional	5.28	6.14	96.48 ***	5.37 ^2^	5.57 ^2^	5.88 ^1^	5.88 ^1^	7.32 ***
	(1.72)	(1.68)		(1.68)	(1.76)	(1.80)	(1.75)	
Family	8.08	7.76	7.82 **	8.04 ^1^	8.09 ^1^	7.93 ^1^	7.61 ^2^	16.71 ***
	(1.45)	(1.47)		(1.51)	(1.47)	(1.37)	(1.47)	
Psychosocial maturity								
Self-competence	3.85	3.81	0.43	3.69 ^2^	3.84 ^1^	3.93 ^1^	3.91 ^1^	17.94 ***
	(0.65)	(0.69)		(0.56)	(0.55)	(0.54)	(0.62)	
Social-competence	3.91	3.78	8.90 **	3.93 ^1^	3.89 ^1^	3.83	3.72 ^2^	7.58 ***
	(0.65)	(0.69)		(0.66)	(0.66)	(0.64)	(0.73)	
Empathy	4.05	3.77	94.91 ***	3.92 ^2^	4.01 ^1^	3.94 ^2^	3.83 ^2^	9.13 ***
	(0.55)	(0.58)		(0.55)	(0.55)	(0.59)	(0.65)	
Emotional maladjustment								
Nervousness	2.43	2.32	20.64 ***	2.41	2.40	2.31	2.41	1.30
	(0.66)	(0.61)		(0.63)	(0.65)	(0.65)	(0.64)	
Emotional	2.61	2.49	15.55 ***	2.64 ^1^	2.55	2.49 ^2^	2.52 ^2^	5.24 **
instability	(0.56)	(0.55)		(0.52)	(0.59)	(0.57)	(0.55)	
Hostility	1.78	1.87	7.77 **	1.89 ^1^	1.84 ^a^	1.74 ^2,b^	1.76 ^2^	7.01 ***
	(0.47)	(0.51)		(0.50)	(0.49)	(0.45)	(0.51)	

* *p* < 0.05; ** *p* < 0.01; *** *p* < 0.001; ^#^ α = 0.05; ^1^ > ^2^ > ^3^ > ^4^; ^a^ > ^b^.
